# Practice Environment and Nurse Outcomes by ANCC Designation: Insights From NDNQI

**DOI:** 10.1155/jonm/1772029

**Published:** 2025-12-12

**Authors:** Nicole George, Angela Pascale, Nora E. Warshawsky

**Affiliations:** ^1^ Nursing Center of Excellence Department, Press Ganey Associates LLC, South Bend, Indiana, USA; ^2^ Clinical Research Department, Press Ganey Associates LLC, South Bend, Indiana, USA

## Abstract

**Aims:**

The aim of this study was to compare nurse outcomes and perceptions of the practice environment among Magnet®‐designated, Pathway to Excellence (PTE)–designated, and non‐ANCC hospitals using hospital‐level data from the National Database of Nursing Quality Indicators® (NDNQI®) database.

**Background:**

The nursing workforce is currently facing prolonged burnout, fatigue, and increased levels of turnover, resulting in nurse leaders reexamining what drives experience, safety, and quality of care. Pursuing nursing excellence credentials such as Magnet and PTE represents a strategic effort to address these issues and enhance the nurse practice environments and advance clinical and patient outcomes.

**Methods:**

This study employed a cross‐sectional design to compare nurse outcomes and practice environment perceptions across three hospital groups: Magnet‐designated, PTE‐designated, and non‐ANCC–designated hospitals using 2023 data from the NDNQI® RN Survey data.

**Results:**

The RN Survey data sample included 26 PTE facilities, 69 Magnet organizations, and 157 non‐ANCC–designated hospitals. Significant differences were identified across hospital designation groups in RN reported measures, specifically for both PTE facilities, and Magnet organizations significantly outperformed non‐ANCC–designated hospitals.

**Conclusions:**

Although PTE and Magnet programs have differing paths to designation, both programs emphasize the importance of quality practice environments to support professional nursing practice. Nurse leaders must prioritize maintaining and optimizing healthy work environments.

## 1. Introduction

In today’s ever evolving and increasingly complex healthcare landscape, nurse leaders continue to face persistent challenges related to recruitment and retention. In addition, the nursing workforce is facing prolonged burnout, fatigue, and increased levels of turnover, resulting in nurse leaders reexamining what drives experience, safety, and quality of care [1, 2]. Pursuing nursing excellence credentials such as Magnet and Pathway to Excellence (PTE) represents a strategic effort to enhance the nurse practice environments and advance clinical and patient outcomes [1, 3–5].

The American Nurses Credentialing Center’s (ANCC) Magnet Recognition Program was established in the early 1990s as a response to growing evidence that organizational characteristics influence nurse retention and optimal patient outcomes [6]. Rooted in the original “forces of Magnetism” identified by the American Academy of Nursing in 1983, the program has since evolved into a rigorous framework encompassing five key components: transformational leadership, structural empowerment, exemplary professional practice, new knowledge and innovation, and empirical outcomes [6]. Magnet designation is widely regarded as the gold standard for nursing excellence and is supported by the literature linking designation with nursing‐sensitive indicators, patient experience, registered nurse engagement scores, and an improved overall nurse practice environment [3, 7–9].

PTE designation emphasizes the creation of positive practice environments rooted in nurse well‐being, safety, and leadership accessibility [4]. Launched in 2007 by the ANCC, the PTE program was developed to recognize healthcare organizations that create positive practice environments where nurses are empowered and engaged [10]. The program is structured around six practice standards: shared decision‐making, leadership, safety, quality, well‐being, and professional development [10].

Unlike Magnet, which emphasizes structures, processes, and outcomes, PTE centers on cultivating the foundational elements of the work environment that directly impact nurse engagement and retention [5]. Both programs require applications with written examples of how the organization meets the standards that are reviewed by program‐specific appraisers. The Magnet program then requires a site visit by the program appraisers to validate the findings, while the PTE program requires nurses to complete a 28‐item survey to validate organizations’ adherence to the standards. Although their operational practices differ, they are united in their quest to secure positive environments to elevate professional nursing practice [5].

In this study, we compared performance on critical nurse outcomes and perceptions of the practice environment data across Magnet‐designated, PTE‐designated, and non‐ANCC (i.e., non‐Magnet– and non‐PTE–designated)–designated hospitals to explore a potentially underappreciated dynamic.

### 1.1. Study Aim

The aim of this study was to compare nurse outcomes and perceptions of the practice environment among Magnet organizations, PTE facilities, and non‐ANCC–designated hospitals. By analyzing hospital‐level data from the National Database of Nursing Quality Indicators (NDNQI), this study sought to identify whether organization designation status is associated with variations in RN reported workplace experiences in acute care hospitals.

## 2. Methods

### 2.1. Study Design and Sample

This study employed a cross‐sectional survey design to compare nurse outcomes and practice environment perceptions across three hospital groups: Magnet‐designated, PTE‐designated, and non‐ANCC–designated hospitals using 2023 data from the NDNQI RN Survey data. The NDNQI RN Survey (https://www.pressganey.com/platform/ndnqi/) is comprised of multiple scales to include the Practice Environment Scale (PES) of the nurse work index, missed nursing care, RN assessed quality of care, professional development access, professional development opportunities, and intent to stay. The NDNQI RN Survey is distributed to eligible registered nurses who have been employed for a minimum of 3 months in their current unit in Press Ganey client hospitals and spend at least 50% of their time providing direct patient care. Participation in the survey was anonymous and voluntary with responses aggregated to the hospital level for analysis. The NDNQI data management procedures were reviewed by the Advarra Institutional Review Board (IRB) and deemed exempt from IRB oversight. Hospitals were classified into one of the three designation groups based on their ANCC designation status as of 2023.

### 2.2. Measures

#### 2.2.1. Nurse Reported Outcomes

PES: The quality of the nursing work environment was evaluated using the PES of the Nursing Work Index (PES‐NWI) [11]. This instrument consists of 31 items that measure nurses’ perceptions of their professional work setting across five domains: nurse participation in hospital affairs, nursing foundations for quality of care, nurse manager ability, leadership and support of nurses, staffing resources and adequacy, and collegial nurse–physician relations. Each item is rated on a 4‐point Likert scale ranging from 1 (*strongly disagree*) to 4 (*strongly agree*).

Missed nursing care: Missed nursing care was measured as the average number of required nursing activities that respondents reported as left incomplete during their most recent shift due to time constraints [11]. Example items included documenting nursing care, completing treatments and procedures, coordinating patient care, and administering medications as scheduled. Participants were asked to identify all applicable items from a set of 15 core nursing activities representing key aspects of nursing care delivery. For this study, missed nursing care was calculated as the average hospital‐level score derived from the total reported missed care events.

Retention: Retention was measured as intention to stay, employed in the unit for the next year, reported as the percentage of nurses who plan to remain in direct patient care on the same unit.

RN assessed quality of care*:* Perceived quality of care was evaluated using a single item from the NDNQI RN Survey that asks, “In general, how would you describe the quality of nursing care delivered to patients on your unit?.” Response options are rated on a four‐point scale: “poor (1),” “fair (2),” “good (3),” and “excellent (4).” This single‐item indicator of nurse‐assessed care quality has been widely applied in nursing research and validated in multiple studies examining unit‐level quality outcomes [12].

Professional development opportunity: Professional development opportunities were measured using a three‐item scale in the NDNQI RN survey to determine if professional development opportunities are offered. Each item is rated on a 6‐point Likert scale ranging from 1 (*strongly disagree*) to 6 (*strongly agree*). The composite score was calculated as the mean of three items.

Professional development access: Professional development access was measured using a three‐item scale in the NDNQI RN survey to determine if professional development opportunities are accessible. Each item is rated on a 6‐point Likert scale ranging from 1 (*strongly disagree*) to 6 (*strongly agree*). The composite score was calculated as the mean of three items.

### 2.3. Data Analysis

Descriptive statistics were used to summarize hospital characteristics and outcome measures across the three designation groups. One‐way analysis of variance (ANOVA) using IBM SPSS Statistics (Version 29) was conducted to examine group differences for each PES domain and nurse‐reported outcome. When significant differences were identified, post hoc tests were performed to determine specific between‐group differences. All analyses were based on annual hospital‐level aggregated data.

## 3. Results

The RN Survey data sample included 27 PTE facilities, 108 Magnet organizations, and 197 non‐ANCC–designated hospitals. Descriptive characteristics, including bed size, teaching status, and case mix, are presented in Table 1.

**Table 1:** Characteristics of participating hospitals by ANCC designation status.

	Pathway	Magnet	Non‐ANCC
Number of hospitals	27	108	197
Bed size			
< 100	16	15	80
100–199	6	31	55
200–299	1	24	24
300–399	2	17	16
400–499	—	7	7
≥ 500	1	13	13
Teaching status			
Academic medical center		11	27
Nonteaching	19	41	108
Teaching	8	55	61
Case mix			
Low	13	31	45
Medium low	6	36	55
Medium high	—	21	3

Significant differences were identified across hospital designation groups in RN reported measures (Table 2). Effect sizes (η^2^) for the ANOVA tests ranged from 0.03 to 0.09, indicating predominantly moderate effects across nurse outcomes. PTE facilities reported significantly higher scores than non‐ANCC–designated hospitals in all 5 domains of the practice environment, including nurse leadership (*p* = 0.007), staffing and resources (*p* < 0.001), foundations for quality of care (*p* < 0.001), nurse participation (*p* < 0.001), nurse–physician relationship (*p* = 0.022), and the overall PES score (*p* = 0.002). Compared to non‐ANCC–designated hospitals, PTE facilities also had higher RN retention (*p* < 0.001), reported less missed nursing care (*p* < 0.001), and reported higher scores in professional development opportunities (*p* < 0.001), professional development access (*p* < 0.001), and RN‐assessed quality of care (*p* < 0.001).

**Table 2:** RN survey outcomes by ANCC designation status.

Outcome measure	PTE (mean ± SD)	Magnet (mean ± SD)	Non‐ANCC (mean ± SD)	F‐statistic	*p* value	Effect size (η^2^) 95% CI	Post hoc (*p* < 0.05)
PES total score	3.23 ± 0.22	3.10 ± 0.17	3.08 ± 0.18	6.51	0.002	0.056 [0.009, 0.118]	Pathway > Magnet; Pathway > non‐ANCC
Nurse leadership	3.32 ± 0.22	3.20 ± 0.15	3.20 ± 0.19	5.12	0.007	0.044 [0.004, 0.102]	Pathway > Magnet; Pathway > non‐ANCC
Staffing and resources	3.04 ± 0.32	2.84 ± 0.23	2.83 ± 0.27	7.61	< 0.001	0.051 [0.010, 0.105]	Pathway > Magnet; Pathway > non‐ANCC
Foundations in QoC	3.29 ± 0.18	3.16 ± 0.16	3.14 ± 0.17	9.44	< 0.001	0.063 [0.016, 0.120]	Pathway > Magnet; Pathway > non‐ANCC
Nurse participation	3.14 ± 0.21	2.98 ± 0.20	2.94 ± 0.22	8.90	< 0.001	0.075 [0.018, 0.143]	Pathway > Magnet; Pathway > non‐ANCC
Nurse–phys. relationship	3.34 ± 0.19	3.25 ± 0.14	3.23 ± 0.17	3.87	0.022	0.034 [0.000, 0.087]	Pathway > non‐ANCC
Retention	83.71 ± 8.77	79.30 ± 6.91	73.88 ± 12.31	18.45^a^	< 0.001	0.089 [0.037, 0.148]	Pathway > non‐ANCC; Magnet > non‐ANCC
Prof dev opportunity	4.87 ± 0.31	4.65 ± 0.32	4.50 ± 0.38	16.91^a^	< 0.001	0.092 [0.036, 0.155]	Pathway > Magnet; Pathway > non‐ANCC; Magnet > non‐ANCC
Prof dev access	4.78 ± 0.28	4.58 ± 0.30	4.51 ± 0.32	8.57	< 0.001	0.055 [0.013, 0.108]	Pathway > Magnet; Pathway > non‐ANCC
RN‐assessed QoC	3.61 ± 0.18	3.56 ± 0.15	3.49 ± 0.20	9.39^a^	< 0.001	0.052 [0.013, 0.102]	Pathway > non‐ANCC; Magnet > non‐ANCC
Missed nursing care	1.13 ± 0.53	1.32 ± 0.52	1.69 ± 0.73	18.81^a^	< 0.001	0.092 [0.038, 0.151]	Pathway < non‐ANCC; Magnet < non‐ANCC

*Note:* Only significant pairwise comparisons (*p* < 0.05) are reported. Prof = professional; Dev = development; Phys. = physician.

Abbreviation: QoC = quality of care.

^a^Welch ANOVA used to test for unequal variances. Post hoc comparisons conducted using Games ‐Howell.

Magnet organizations similarly outperformed non‐ANCC–designated hospitals across multiple RN reported outcomes. Compared to non‐ANCC–designated hospitals, Magnet organizations reported significantly higher scores for professional development opportunities (*p* < 0.001), RN assessed quality of care (*p* < 0.001), and retention (*p* < 0.001), along with lower levels of missed nursing care (*p* < 0.001).

In comparisons between the two ANCC‐designated groups, PTE facilities reported significantly higher scores than Magnet‐designated organizations in 4 of the 5 domains of the practice environment, including nurse leadership (*p* = 0.007), staffing and resources (*p* < 0.001), foundations for quality of care (*p* < 0.001), nurse participation (*p* < 0.001), and the overall PES score (*p* = 0.002), with the exception of nurse–physician relationships. In addition, PTE facilities reported significantly higher scores than Magnet‐designated organizations in professional development opportunity (*p* < 0.001) and professional development access (*p* < 0.001).

## 4. Discussion

The primary aim of this study was to compare the practice environments and nurse outcomes of PTE facilities, Magnet organizations, and those that are not ANCC‐designated using a national dataset. The findings demonstrate that both PTE facilities and Magnet organizations significantly outperformed non‐ANCC–designated hospitals across a range of nurse‐reported outcomes.

PTE facilities significantly outperformed non‐ANCC–designated hospitals in overall practice environment and in all 5 domains of the practice environment: nurse participation in hospital affairs, foundations for quality care, nurse manager leadership and support, nurse–physician relationship, and adequate staffing and resources. PTE designated facilities also significantly outperformed non‐ANCC–designated hospitals in access to professional development opportunities, RN retention, RN‐assessed quality of care, and reduced missed care. Nurses working in PTE facilities also scored their work environments higher than nurses working in Magnet organizations across all domains. Moreover, PTE facilities significantly outperformed Magnet organizations in 4 of the 5 practice environment domains (with the exception of nurse–physician relationships), as well as higher professional development access and opportunity. More importantly, PTE facilities achieved significantly better nurse outcomes, higher levels of retention, quality of care, and fewer missed nursing care activities, than nurses working in non‐ANCC–designated hospitals. Notably, PTE nurses achieved 10% higher nurse retention than nurses working in non‐ANCC–designated hospitals. Overall, these findings are consistent with the six standards of the PTE model and demonstrate its effectiveness in retaining nurses.

Nurses working in Magnet organizations scored their work environments higher than nurses working in non‐ANCC–designated hospitals. Although the raw scores were descriptively higher than non‐ANCC–designated hospitals, they were only significantly higher for professional development opportunity and nurse outcomes of retention, nurse‐assessed quality of care, and lower missed nursing care. On the one hand, this finding disrupts conventional assumptions about Magnet organizations in comparison to non‐Magnet hospitals. A plausible explanation for this observation may be rooted in the Magnet requirements. Organizations seeking Magnet designation need to outperform benchmarked mean scores, but there is no requirement for statistical outperformance. This does not mean that the Magnet designation lacks value. Magnet organizations still significantly outperformed non‐ANCC–designated hospitals in critical nurse outcomes of higher retention, higher RN‐assessed quality of care, and lower missed nursing care. Magnet’s evidence‐based model with emphasis on empirical nurse and patient outcomes was demonstrated in this analysis.

Although the PTE facilities and Magnet organizations outperformed the non‐ANCC–designated hospitals in this study, the scores for each domain were generally greater than 3.0 and very similiar across hospital types. This finding suggests that this sample of non‐ANCC–designated hospitals performed at a high level. This might reflect that Press Ganey clients are seeking nursing excellence even if they forgo the ANCC recognition, or they might be “on the journey” to apply for one of these ANCC designations [2]. It may also reflect the work of the growing cadre of DNP‐prepared nurse leaders who are implementing well‐established evidence‐based strategies to recruit and retain their nurses [13, 14]. While our study revealed several meaningful differences across designation types, the RN‐physician relationship domain did not yield statistically significant variation among Magnet, PTE, and non‐ANCC–designated hospitals. Of note, the mean RN–physician relationship scores ranged from 3.25 to 3.34, indicating a solid “good” score. Although the evidence suggests better interprofessional collaboration is needed, it is possible that RN–physician collaboration and communication processes and initiatives have been hardwired across the spectrum following recommendations from the National Academy of Medicine (known then as the Institute of Medicine) [15–17].

Both programs emphasize the importance of quality practice environments to support professional nursing practice [5]. The two programs differ in their validation processes to ensure that the relevant standards are meaningfully embedded in their hospital culture and daily practice. The PTE programs have nurses complete a survey to affirm the presence of the PTE elements, whereas the Magnet program validation is through Magnet appraiser site visits. Another critical difference is that the PTE program is focused on the structures and processes to achieve transparent communication, well‐being, quality, and safety [6, 10]. The Magnet program emphasizes transformational leadership, structural empowerment, and innovation to achieve quality outcomes. We will not go as far as recommending one program over the other; rather, we encourage nurse leaders to critically evaluate how each of these programs aligns with their organization’s nursing strategic plans.

The moderate effect sizes observed across hospital designation groups highlight that Magnet and PTE recognition meaningfully contribute to improved nurse outcomes and practice environment perceptions. At the same time, the moderate magnitude of these effects indicates that broader organizational influences remain important in shaping nurses’ experiences beyond designation status.

### 4.1. Implications for Nurse Leaders

The consistently high nurse‐reported outcomes of the practice environment across hospital designation groups highlight the critical importance of maintaining and advancing a healthy work environment. Decades of research have established that healthy work environments, built on foundations such as effective communication, collaborative relationships, adequate staffing and supportive leadership, are strongly linked to higher nurse satisfaction, retention, and patient care outcomes [11]. Nurse leaders play a central role in sustaining these environments by implementing evidence‐based strategies that reinforce these core elements. This responsibility is becoming increasingly urgent given evolving workplace expectations. While nurses across generations prioritize supportive and safe practice environments, Generation Z nurses, in particular, are less willing to tolerate unhealthy or unsupportive work environments [18]. As this generation becomes a larger portion of the nursing workforce in the coming years, the expectation for healthy work environments evolves from a best practice to a generational standard, making it a strategic priority for nurse leaders seeking to sustain engagement and retention.

### 4.2. Limitations

Several limitations should be considered when interpreting the findings of the study. First, the sample was limited to hospitals participating in NDNQI, which includes organizations that voluntarily engage in national benchmarking efforts and are often focused on quality improvement. As such, the full range of non‐ANCC–designated hospitals, particularly those with fewer resources or different operational priorities, may not be fully represented, potentially limiting the generalizability of the study findings. Second, the study utilized a cross‐sectional design, which limits the ability to examine longitudinal trends or account for historical factors that may influence RN‐reported outcomes, such as national initiatives to improve work environments, increase the proportion of BSN‐prepared nurses at the bedside, and expand the influence of DNP‐prepared nurse leaders promoting evidence‐based practice. Third, a ceiling effect in the outcome measures may have resulted from widespread improvements in nursing practice environments, resulting in consistently high scores and limited variability that reduced the ability to detect meaningful differences between organizational designations. Lastly, the study could not distinguish between non‐ANCC–designated hospitals actively seeking Magnet or PTE designation and those not engaged in the process, limiting the ability to assess how organizational investment in the ANCC designation journey may influence nurse‐reported outcomes.

## 5. Conclusion

Nursing has a strong foundation of evidence on the structures and processes needed to create environments that drive professional nursing practice. The ANCC recognition programs codified the essential elements to achieve critical nurse outcomes. The overall success of each programs’ outcomes has fueled the impetus for organizations to earn these prestigious designations. The journey can be challenging, but the good news is that nursing has a growing cadre of experts to translate and implement the strategy. Retaining more nurses in clinical practice is the true measure of success as nurse leaders embark on the journey to nursing excellence.

## Disclosure

The findings and conclusions presented in this report are based on the authors′ independent analysis and do not represent the official stance of AN Enterprise, Magnet, PTE, or any affiliated organizations.

## Conflicts of Interest

The authors declare no conflicts of interest.

## Funding

The authors received no specific funding for this study.

## Data Availability

The NDNQI data used in this study are proprietary and maintained by Press Ganey. Because the database includes sensitive, institution‐level quality and workforce information submitted under contractual agreements, it is not publicly available. Access is restricted to participating healthcare organizations in accordance with data use and confidentiality policies.
